# Focus on the sex-specific neural markers in the discrimination of various degrees of depression

**DOI:** 10.1093/psyrad/kkae006

**Published:** 2024-04-09

**Authors:** Yaqin Li, Xinyu Yan, Xianxin Meng, Jiajin Yuan

**Affiliations:** Sichuan Key Laboratory of Psychology and Behavior of Discipline Inspection and Supervision, Institute of Brain and Psychological Sciences, Sichuan Normal University, Chengdu 610066, China; Psychologie, Sciences de l'Education et Logopédie, Université libre de Bruxelles (ULB), Brussels 1050, Belgium; School of Psychology, Fujian Normal University, Fuzhou 350117, China; Sichuan Key Laboratory of Psychology and Behavior of Discipline Inspection and Supervision, Institute of Brain and Psychological Sciences, Sichuan Normal University, Chengdu 610066, China

## The gender difference of MDD in epidemiology and clinical symptomatology

Major depressive disorder (MDD) is a common and serious mental disorder (Li *et al*., 2022; Malhi & Mann, 2018). Male and female patients with MDD have shown some differences in the epidemiology and clinical symptomatology. Specifically, women have higher risk of depression and prevalence rates than men (Angst *et al*., 2002; Zhang *et al*., 2023), even among adolescents (Daly, 2022). Meanwhile, males with MDD experience less severe sleep disturbance than females with MDD (Yang *et al*., 2017), while females with MDD have a better response to antidepressants than males with MDD (Sramek *et al*., 2016). Additionally, female MDD patients are more likely to have suicidal thoughts, whereas male MDD patients are more likely to successfully kill themselves (Cavanagh *et al*., 2017).

## Comments on the study by Mou *et al*. (2023)

Mou *et al*. (2023) conducted a study with 61 patients with MDD and an equal number of healthy controls (both comprising 36 females and 25 males). Using magnetic resonance imaging (MRI), the researchers found that male MDD patients exhibited thinner cortical thickness (CT) in the right precentral region compared to female MDD patients (not significant after Bonferroni correction), whereas female MDD patients exhibited reduced surface area (SA) in specific brain regions, including the right superior frontal, medial orbitofrontal gyrus, inferior frontal, gyrus triangle, superior temporal, middle temporal, lateral occipital gyrus, and inferior parietal lobule, when compared with male MDD patients (after Bonferroni correction). Additionally, the SA of the right superior temporal, middle temporal, and lateral occipital gyrus in female MDD patients was correlated with illness duration; however, this correlation was not observed in male MDD patients (Mou *et al*., 2023).

This finding demonstrated that sex differences existed in the brain cortical structure of MDD, which provided evidence for sex differences in MDD and contributed to enhancing the effectiveness of targeted therapy for MDD. Some researchers found that male MDD patients exhibited selectively decreased gray matter volume (GMV) in the medial prefrontal gyrus, while female MDD displayed selectively decreased GMV in the dorsal medial prefrontal gyrus (Yang *et al*., 2017). Others found that compared with female MDD patients, male MDD patients exhibited decreased amplitude of low-frequency fluctuation (ALFF) in the bilateral caudate nucleus and posterior cingulate gyrus (Mei *et al*., 2022). However, in MDD, fewer researchers have paid attention to sex-specific brain structure alterations in the SA and CT. The findings of Mou *et al*.’s (2023) study further demonstrate the difference that occurred in the SA and CT of certain brain regions, expanding previous findings. Therefore, Mou *et al*. (2023) have contributed to the field of affective neuroscience.

It is well known that structural brain abnormalities in MDD primarily include the prefrontal cortex, cingulate cortex, hippocampus, occipital cortex, partial cortex, and grey matter (Chen *et al*., 2017; Korgaonkar *et al*., 2014; Liu *et al*., 2022; Li *et al*., 2023). Specifically, when compared with healthy controls, MDD patients mainly exhibited lower efficiency in the default-mode network (e.g. hippocampus, superior parietal lobule) for transferring information, grey matter morphological networks were disrupted (Chen *et al*., 2017) and the directed connectivity from the parietal-to-central region was disrupted (Liu *et al*., 2022). What is more, Mou *et al*. (2023) indicated the significant brain cortical structure differences in SA between male MDD and female MDD groups, and these brain regions mainly in default-mode network, frontoparietal network (right inferior frontal gyrus triangle, superior frontal, medial orbitofrontal gyrus, superior temporal, middle temporal gyrus, and inferior parietal lobule), and visual network (right lateral occipital gyrus). They further expanded previous results, demonstrating the sex-specific brain structure in MDD.

## Implications and future directions

Based on a study by Mou *et al*. (2023), what we should do in the future? First, the cause of sex differences in MDD has not been concluded. Some researchers speculate that the difference may stem from sex-specific genes (e.g. sex-genetic susceptibility), sex hormones, or different clinical symptoms (Hu *et al*., 2022; Mei *et al*., 2022; Mou *et al*., 2023), yet, no study has found how these sex-specific mechanisms affect the brain structure and development of MDD. Thus, addressing this gap is crucial for future research. It is worth noting that numerous animal model studies have extensively revealed potential sex-specific mechanisms of MDD (Eid *et al*., 2019; Hao *et al*., 2019; Ma *et al*., 2019; Palanza, 2001), including serotonin, the hypothalamic–pituitary–adrenal axis, brain-derived neurotrophic factor, etc. Therefore, we can further explore how these sex-specific mechanisms affect the whole development of MDD by comparing the similarities and differences between animal model experiments and human MDD experiments. This will not only address the cause of sex differences in MDD, but also help the future development of gender specific interventions and clinical treatments.

Second, this study exclusively focused on MDD and neglected subclinical and high-risk individuals. To comprehend the complete picture of sex differences in depression, we should explore the characteristics at different stages of depression using machine learning. Specifically, employing machine learning techniques such as support vector machine learning or support vector regression can help classify sex-specific neural structure characteristics in individuals with various degrees of depression (e.g. subclinical, high-risk, and MDD individuals). Subsequently, utilizing neural data to identify participants' gender will enable the formation of neural patterns for subclinical, high-risk, and MDD individuals, offering improved criteria for diagnosing different degrees of depression. Additionally, it is necessary to adopt longitudinal studies to explore the developmental and pathophysiological mechanisms by which MDD differs in male and female patients.

Meanwhile, we should focus on the effect of inflammation on sex-specific depression. Although some findings have found that inflammatory markers (e.g. high sensitivity C-reactive protein, white blood cells) increased with increasing severity of symptoms of depression, this correlation is affected by gender (Shafiee *et al*., 2017; Tabatabaeizadeh *et al*., 2018). Importantly, this is not a direct cause-and-effect relationship. Moreover, researchers have found that depression treatments (e.g. transcranial magnetic stimulation, electroconvulsive therapy) also exited gender difference (Bloch *et al*., 2005; Sackeim *et al*., 2020; Tik, 2023). In light of this, we can combine sex-specific inflammatory and neural markers to enhance the effectiveness of targeted therapy for MDD, thus improving clinical treatments.

## Conclusions

In conclusion, Mou *et al*.’s (2023) findings indicated that there were differences in the brain cortical structure between males and females, which were characterized by the brain cortical structure’s SA and CT. It helps to better understand the pathogenesis of MDD and enhance the effectiveness of targeted therapy for MDD. Moving forward, exploring sex characteristics and their causes at different stages of depression (subclinical, high-risk, and MDD individuals) through the integration of machine learning with longitudinal studies is essential. Additionally, it is important to pay attention to gender differences in inflammation and treatments in MDD. By combing sex-specific inflammatory and neural markers further guide gender specific interventions and clinical treatments in depression. Only in this way can we comprehend the complete picture of sex differences in depression and advance targeted therapy for MDD.
